# Swelling and Helium Bubble Morphology in a Cryogenically Treated FeCrNi Alloy with Martensitic Transformation and Reversion after Helium Implantation

**DOI:** 10.3390/ma12172821

**Published:** 2019-09-02

**Authors:** Feifei Zhang, Lynn Boatner, Yanwen Zhang, Di Chen, Yongqiang Wang, Lumin Wang

**Affiliations:** 1 Department of Nuclear Engineering and Radiological Sciences, University of Michigan, Ann Arbor, MI 48108, USA; 2 Materials Science and Technology Division, Oak Ridge National Laboratory, Oak Ridge, TN 37831, USA; 3 Department of Materials Science and Engineering, University of Tennessee-Knoxville, Knoxville, TN 37996, USA; 4 Materials Science and Technology Division, Los Alamos National Laboratory, Los Alamos, NM 87545, USA

**Keywords:** FeCrNi alloy, helium bubble, bubble swelling, ion irradiation, phase transformation

## Abstract

A cryo-quenched 70 wt % Fe-15 wt% Cr-15 wt% Ni single-crystal alloy with fcc (face centered cubic), bcc (body centered cubic), and hcp (hexagonal close packed) phases was implanted with 200 keV He^+^ ions up to 2 × 10^17^ ions·cm^−2^ at 773 K. Surface-relief features were observed subsequent to the He^+^ ion implantation, and transmission electron microscopy was used to characterize both the surface relief properties and the details of associated “swelling effects” arising cumulatively from the austenitic-to-martensitic phase transformation and helium ion-induced bubble evolution in the single-crystal ternary alloy. The bubble size in the bcc phase was found to be larger than that in the fcc phase, while the bubble density in the bcc phase was correspondingly lower. The phase boundaries with misfit dislocations formed during the martensitic transformation and reversion processes served as helium traps that dispersed the helium bubble distribution. Swelling caused by the phase transformation in the alloy was dominant compared to that caused by helium bubble formation due to the limited depth of the helium ion implantation. The detailed morphology of helium bubbles formed in the bcc, hcp, and fcc phases were compared and correlated with the characters of each phase. The helium diffusion coefficient under irradiation at 773 K in the bcc phase was much higher (i.e., by several orders of magnitude) than that in the fcc phase and led to faster bubble growth. Moreover, the misfit phase boundaries were shown to be effective sites for the diffusion of helium atoms. This feature may be considered to be a desirable property for improving the radiation tolerance of the subject, ternary alloy.

## 1. Introduction

FeCrNi alloys are often considered as model austenitic stainless steels, i.e., systems that are important structural materials for use in present-day and future nuclear reactors. In particular, such materials that are able to withstand extreme irradiation doses are of general importance for applications in advanced nuclear-energy systems [1,2].

Macroscopic radiation damage effects in structural components of nuclear devices, such as fission or fusion reactors, are the consequence of two fundamentally different types of interactions between the energetic particles in the irradiation process and the atoms in the material lattice, i.e., atomic displacements result in vacancy and self-interstitial type lattice defects and nuclear reactions result in the introduction of foreign elements. The defect agglomerates that are produced contribute to swelling, hardening, amorphization, and embrittlement, i.e., properties that may accelerate the materials’ degradation and potentially lead to failure. In particular, the (n, α) transmutation reaction caused by neutron irradiation produces large amounts of helium atoms that may lead to the precipitation and formation of helium bubbles [3,4]. This creation of helium atoms in metals is considered to be of particular concern, since their precipitation into large bubbles can substantially deteriorate the mechanical properties of the material [5].

Different strategies continue to be explored to enhance the irradiation tolerance of metals and alloys, including the tolerance of the materials to helium build-up during neutron irradiation. In particular, the introduction of a high density of grain boundaries and interphase boundaries is one approach for increasing the radiation resistance that is currently being explored [6,7,8,9,10]. Most grain boundaries and interfaces are effective sinks for point defects that can potentially lead to enhanced irradiation tolerance. Atomistic simulations have, in fact, confirmed the ability of grain boundaries to remove collision cascade-induced point defects in both fcc and bcc metals [11,12]. Additionally, studies of multilayered composites have shown that heterophase interfaces are also excellent point defect sinks [13].

Recently, Boatner et al. have reported that large-size single crystal Fe-15Cr-15Ni alloys can be used as a decorative steel after the austenitic-to-martensitic phase transformation is induced by a cryo-quenching method [14]. Extensive research has also been carried out previously on the formation of bcc α′-martensite and hcp ε-martensite in Fe-Cr-Ni system alloys [15,16,17,18]. For high-temperature applications, the reverse transformation of these phases to γ-austenite should be considered. This transformation is of interest both in structural mechanics and radiation resistance considering the high density of dislocations and phase boundaries formed during the transformation. According to previous researchers, phase reversion is also significant in relation to the behavior of certain stainless steels used in reactor applications [19]. The formation and co-existence of α′-martensite, hcp ε-martensite, and γ-austenite phases in FeCrNi alloys (along with their different radiation behavior) suggest that it may be possible to markedly improve the irradiation resistance of many materials by designing or controlling the composition of the constituent phases. 

In the present work, we report the properties of the helium bubble evolution in cryo-quenched single crystal Fe-15Cr-15Ni alloys after He^+^ irradiation at 773 K. Here, we provide evidence that radiation tolerance can be improved by misfit phase boundaries between the different phases in the Fe-15Cr-15Ni alloy. Specifically, the typical features of helium bubbles formed in the fcc and bcc phases were examined in detail and correlated with the characteristics of each phase.

## 2. Experimental

The Fe-Cr-Ni alloy specimen used here was produced by first growing a single crystal of a 70 wt % Fe, 15 wt% Cr, 15 wt% Ni composition, referred to as Fe-15Cr-15Ni in this work. High-purity elemental components (e.g., 99.99% to 99.999% pure Fe, Ni, and Cr) were used to form the alloy. Laue back-reflection X-ray methods were used to orient Fe-15Cr-15Ni austenitic alloy single crystals that were then cut using an electric-arc discharge machine to expose the desired crystal surface. These surface cuts were chosen perpendicular to the (100), (110), and (111) planes of the different single crystals, respectively. After shaping of the item that was to be produced by the metal-removal method, the work piece with a crystallographically oriented surface was lapped and then given a final high polish. The material was then cryogenically quenched at 77 K by immersion in liquid nitrogen followed by a return to room temperature. Cryogenic quenching results in the formation of a surface relief on the ternary single-crystal alloy. Additional details on processing this alloy are available elsewhere [14]. 

The crystal samples with different surface orientations were affixed on a copper block using a high-temperature silver paste. A thermocouple was attached to the block to monitor the sample temperature during the He^+^ irradiation. The crystals were then irradiated with 200 keV He^+^ ions at 773 K to a fluence of 2 × 10^17^ ions cm^−2^ at a flux of approximately 2 × 10^13^ ions cm^−2^·s^−1^ using the Danfysik Research Ion Implanter (Danfysik A/S, Taastrup, Denmark) at the Ion Beam Materials Laboratory in Los Alamos National Laboratory. During the irradiation process, the target chamber was maintained at a typical vacuum level of 10^−7^ Torr. The irradiation-induced damage profile and helium concentration were predicted using the Stopping and Range of Ions in Matter (SRIM) code by choosing the calculation type “Ion distribution and quick calculation of damage” [20,21]. The composition and density of the target in the calculation was the Fe-15Cr-15Ni alloy with a density of 7.987 g/cm^3^. Likewise, a value of 40 eV was set for the displacement energy threshold energy for Fe, Cr, and Ni [22]. 

The irradiated samples were analyzed using transmission electron microscopy (TEM) and scanning transmission electron microscopy (STEM) in cross-section. All of the TEM samples were prepared by focused ion beam (FIB) lift-out techniques. The TEM samples were examined using JEOL 3011 (JEOL, Tokyo, Japan) and JEOL 3100RS (JEOL, Tokyo, Japan) transmission electron microscopes operated using 300 keV electrons. The helium bubbles formed during the irradiation process were imaged by conventional TEM with a Fresnel contrast mechanism, and the crystallographic structure was analyzed by high-resolution TEM (HRTEM) and selected area diffraction (SAD) techniques.

## 3. Results

### 3.1. Surface Morphology after Helium Irradiation at 773 K

**Figure 1:**
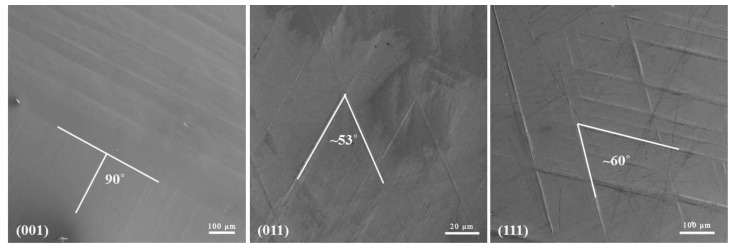
Optical micrographs of the surface morphology of the cryo-quenched Fe-15Cr-15Ni single-crystal alloy after He^+^ irradiation to 2 × 10^17^ ions cm^−2^ at 500 °C (irradiated surfaces are well polished prior to He^+^ irradiation).

After He^+^ irradiation at 773 K, a relief pattern formed on the surface of the Fe-15Cr-15Ni single crystal. SEM results in Figure 1 show the surface relief patterns formed on {001}, {011}, and {111} surfaces. Since the samples were polished before the He^+^ ion irradiation, the surface-relief features after He^+^ ion irradiation may be induced by either a phase transformation at the elevated temperature during the implantation or helium bubble swelling or a combination of both. Similar structures were also reported by Breedis [23], in grains of polycrystal of a Fe-16Cr-12Ni alloy (previously quenched to 173 K) when annealing the samples at 673 to 1173 K.

### 3.2. Microstructural Observations of γ-Austenite, α′-Martensite, and ε-Martensite

The decorative surface structures produced from cooling an Fe-15Cr-15Ni alloy single crystal have been reported previously by Boatner et al. [14] as consisting primarily of two differing crystalline phases (i.e., austenite and retained martensite) after cycling through the phase transition. In Fe-15Cr-15Ni austenitic stainless steels, two martensitic phases form after cooling below Ms. temperature (the martensite start temperature). The ε martensitic phase has an hcp structure and forms as plates, while α′ martensite with a bcc structure forms as laths having a <110>_γ_ long direction, lying in bands bounded by {111}_γ_ planes [23,24].

Helium irradiation was performed at 773 K, which means the samples were effectively undergoing heat treatment that might induce some phase alteration effects (e.g., a martensite reverse transformation). Various microstructural features of reversed γ have been reported [24,25,26], including stacking faults, fine twins, and areas with a high dislocation density and subgrains. 

**Figure 2:**
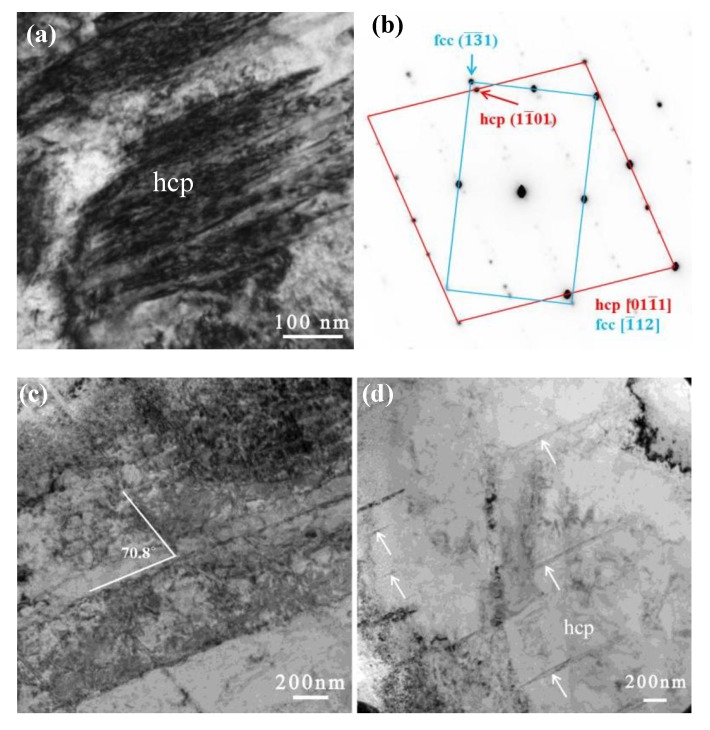
TEM images of the microstructure of the Fe-15Cr-15Ni alloy cryogenically treated at 77 K and heated to 773 K for a 2 × 10^17^ ions cm^−2^ He^+^ irradiation—showing ε martensite in the matrix. (**a**) ε-martensite (hcp phase) and (**b**) corresponding electron diffraction pattern, (**c**) and (**d**) nano-layers of ε-martensite.

**Figure 3:**
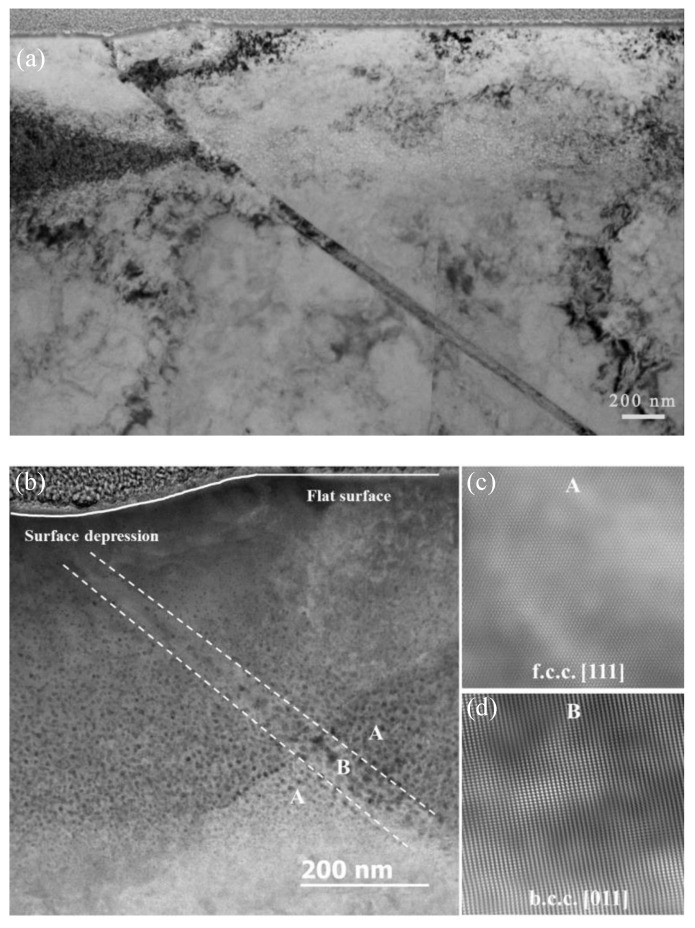
TEM images of the microstructure of the Fe-15Ni-15Cr alloy cryogenically treated at 77 K and heated to 773 K for a 2 × 10^17^ ions cm^−2^ He^+^ irradiation—showing α′ martensite lath that formed with the <110> direction. The surface depression is related to the martensitic reverse transformation. (**a**) TEM micrograph showing a martensite lath in matrix, (**b**) STEM micrograph show helium bubbles (black dots) in martensite lath and matrix, (**c**) and (**d**) is inversed FFT showing fcc and bcc structure of martensite lath and matrix.

Figure 2 and Figure 3 show results for TEM samples that were lifted out from the surface relief and depression regions, respectively. Figure 2 shows an area containing hcp regions. Figure 2a shows the hcp phase at one of its zone axes, and Figure 2b shows the corresponding electron diffraction pattern. Figure 2c,d show the most common morphology of the ε-martensite phase, that is a nano-layer hcp phase in this case. Figure 3a shows an area containing bcc regions, with a lath-like band crossing over the entire cross-section of the sample. The crystallographic relationships between the austenite (γ) and bcc phases were examined in the electron microscope by placing an appropriate diffraction aperture across a common boundary to allow the superposition of both grain orientations in the same selected area diffraction (SAD) pattern (Figure 3a). The results are exemplified in the composite shown in Figure 3 that illustrates the following orientation relationships: {111}_γ_//{011}_α′_, [101]_γ_//[111]_α_. The fcc/bcc orientation relationships were consistent along with the Kurdjumov–Sachs (K–S) relationship. Additional evidence via high-resolution TEM (HRTEM) is shown in Figure 3c,d, which confirms the fcc and bcc structure.

### 3.3. Irradiation Damage of Cryo-Quenched Fe-15Cr-15Ni Alloy

**Figure 4:**
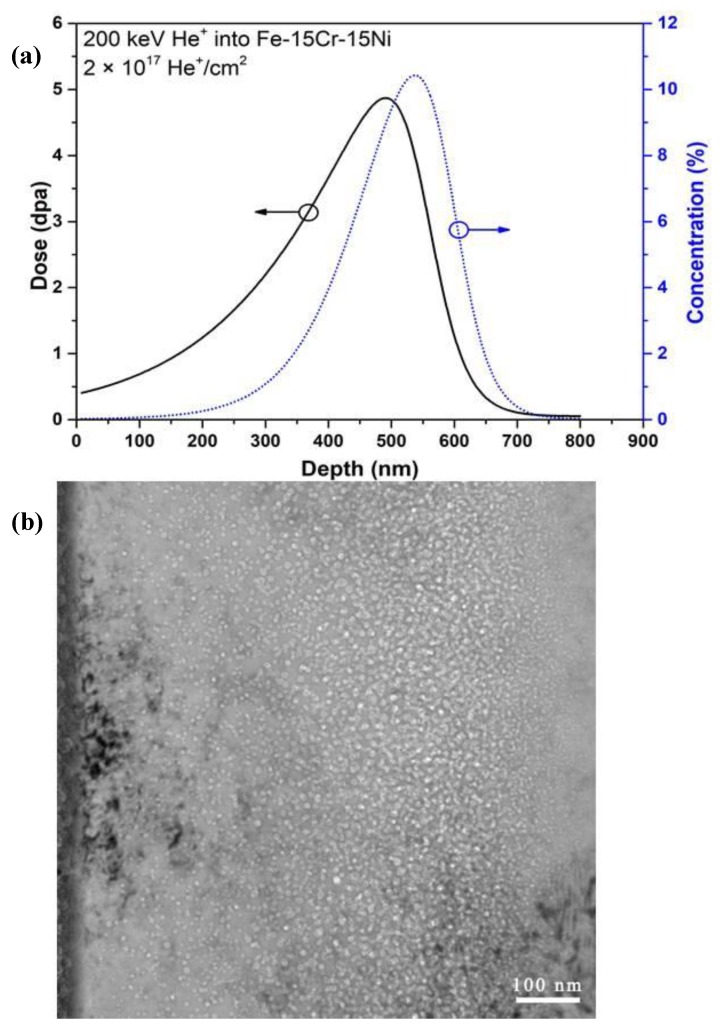
(**a**) The damage distribution in dose (dpa) and helium concentration for 200 keV He^+^ irradiation to 2 × 10^17^ ions cm^−2^, as predicted by SRIM. (**b**) A bright-field Fresnel-type image recorded in an under-focused condition where the bubbles are shown as bright regions.

The dose and helium concentration as a function of sample depth, as predicted by SRIM for the sample irradiated to a fluence of 2 × 10^17^ ions cm^−2^, are shown in Figure 4a. The dose shows the predicted damage profile extending from the surface to a depth of 700 nm with a peak damage of ~5 dpa at 500 nm. The predicted helium concentration profile reveals a relatively sharp peak, extending from 400 to 700 nm with a peak of 1 × 10^5^ appm of helium at a depth of 550 nm.

Cross-section TEM micrographs recorded in “under-focused” conditions for the sample irradiated to 2 × 10^17^ ions cm^−2^ are shown in Figure 4b. The resulting Fresnel contrast observed here is indicative of cavities (either bubbles or voids) in the matrix. In this particular material, these cavities are pressurized helium bubbles. The discernible bubbles extend from the surface to a depth of ~800 nm. As expected, the bubbles in the regions of the higher helium concentrations are, on average, larger than those in the lower-concentration regions—although significant variations in the sizes were observed with increasing distance from the implantation surface. Larger bubbles are observed in the center of the band of bubbles, and smaller bubbles are found on either side of these regions.

#### 3.3.1. Helium Bubble Distribution in the Cryo-Quenched Fe-15Cr-15Ni Alloy

Cryo-quenching to 77 K induced a phase transformation in the Fe-15Cr-15Ni alloy single crystal [14]. Diffractometer evidence from single crystals and polycrystalline aggregates shows that three structures (fcc, hcp, bcc) occur in varying proportions. To investigate the helium bubble distribution in cryo-quenched Fe-15Cr-15Ni alloy, samples with [110]_γ_, [100]_γ_, [111]_γ_, and [112]_γ_ zone axes parallel to the incident electron beam were selected.

**Figure 5:**
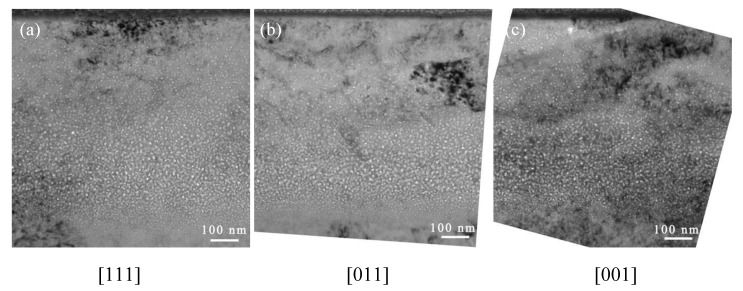
Low magnification of 200 keV He^+^ irradiated Fe-15Cr-15Ni samples with different orientations (**a**) [111], (**b**) [011], (**c**) [001]. No channeling effect was observed.

In order to study the radiation-induced defects as well as the helium bubble formation and growth processes, cross-section TEM characterization of the irradiated bulk materials was carried out. TEM was employed to investigate both the helium bubble morphology and phase transformation. Figure 5 shows the helium bubble distribution and irradiation-induced features at low magnification for samples with the (001)-, (011)-, and (111)-orientations. No channeling effect was observed, and the helium distribution matches the SRIM predictions.

**Figure 6:**
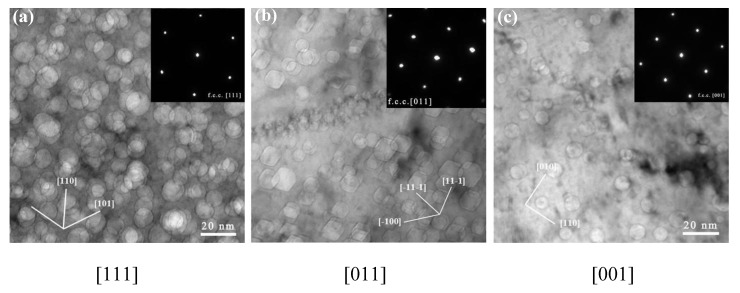
Comparison of the experimental observations of helium bubbles with the projected shape of an octahedron for the different zone axes: (**a**) [111], (**b**) [011], and (**c**) [001] in the fcc matrix.

Due to the high-temperature irradiation, faceted helium bubbles were observed in all of the irradiated samples. Figure 6 shows the details of the faceted helium bubbles at higher magnification. As indicated by the lines and inserted diffraction patterns in Figure 6a–c, although the bubble size distribution is broad, the shape of the bubbles is faceted. There are preferential orientations for the facets (viewed along the zone axis of [011]): The edges of all of the faceted bubbles are parallel to [001], [11–1], or [–11–1].

Based on these observations, a polyhedron composed of {111} planes is suggested to surround the bubble. Geometric construction of a polyhedron consisting of {111} planes shows an octahedral shape. In most cases, a truncated octahedron or octahedron with rounded edges and corners is more energetically favorable [27]. Since it is embedded in the substrate and consists of {111} substrate planes, the octahedron has the same symmetry as the substrate. In order to minimize the overall free energy, it is expected that the facets with the lowest surface energy will occupy most of the surface. In the fcc structure, the lowest surface energy is found on the most closely packed {111} planes. In our case, {100} planes occupy the corners of the octahedral composed of {111} planes, which means that there is a {111} octahedron with six {100} truncations. The total surface area of a faceted bubble with the {111} surface decreases when the bubble is truncated by the {100} planes. A truncated octahedron or rounded corners are more energetically favorable.

**Figure 7:**
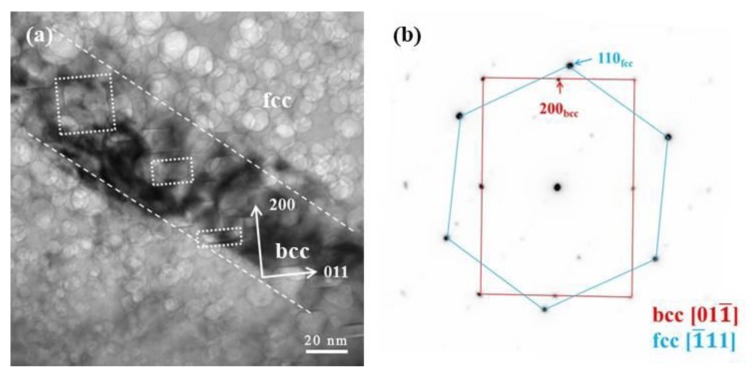
(**a**) Faceted helium bubbles in an α′-martensite lath (bcc phase). The orientation relationship between γ-austenite and α′-martensite was determined by (**b**) the SAD pattern, {111}_γ_//{011}_α′_, [101]_γ_//[111]_α_

Figure 3 represents the process of phase identification from a [111]_γ_ zone axis. As shown in Figure 3a, a long, narrow trace passes through the entire cross-section of the image, illustrating the existence of the α-structure. It shows the α-phase with a thickness of ~20 nm. At higher magnification, as shown in Figure 7a, faceted bubbles in the bcc phase are observed. Figure 7b shows the indexed SAD pattern, indicating that the fcc and bcc phases are associated with the zone axis of [111]_γ_//[011]_α_. The observed orientation relationship is consistent with the well-known K–S (Kurdjumov–Sachs) relationship, i.e., the [111]_γ_//[011]_α_ of fcc and bcc lattices.

Also shown in Figure 7 are the different helium bubble morphologies in the fcc and bcc phases. In the fcc phase, the helium bubbles are somewhat rounded. In the bcc phase, however, the helium bubbles are less rounded and more “plate-like” in (and near) the helium peak area. The facets of the helium bubbles in the bcc phase are composed of {011}_α_ and {001}_α_ planes. 

**Figure 8:**
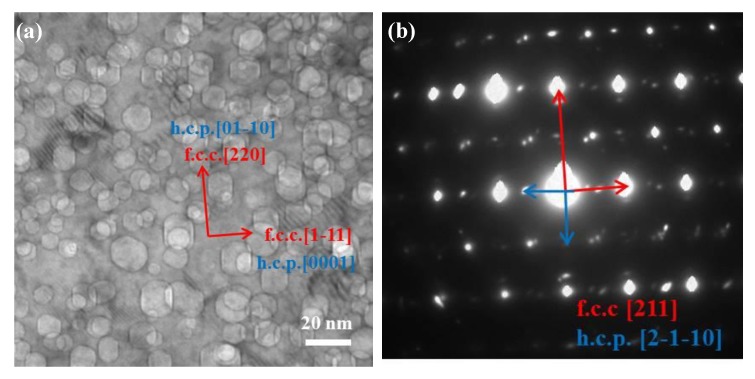
(**a**) TEM micrograph and (**b**) SAED pattern show the evidence of the co-existence of fcc and hcp phases along with helium bubbles in the irradiated area.

The TEM micrographs show narrow (nm wide) traces that are parallel to {111}_γ_ in different irradiated samples with different orientations, i.e., [112] and [011]. Figure 8 is a TEM image that shows the microstructures observed at a depth of 300 nm from the sample surface. Clearly, the BF TEM image and SAD patterns reveal the formation of overlapping fcc and hcp phases. The SAD pattern was recorded by tilting the specimen along the beam direction of [211] γ- austenite. Figure 8b shows the indexed SAD pattern, comprised of fcc phase and hcp phases. The low-energy surfaces in the fcc phase are (111), (100), and (011), and the low energy surfaces in the hcp phase are (01–11), (0001), (01–10), and (11–20). Faceted helium bubbles are observed with two different morphologies, following the minimized energy rule in the fcc and hcp phases.

#### 3.3.2. Helium Bubble-Induced Swelling and Phase Transformation-Induced Surface Relief

**Figure 9:**
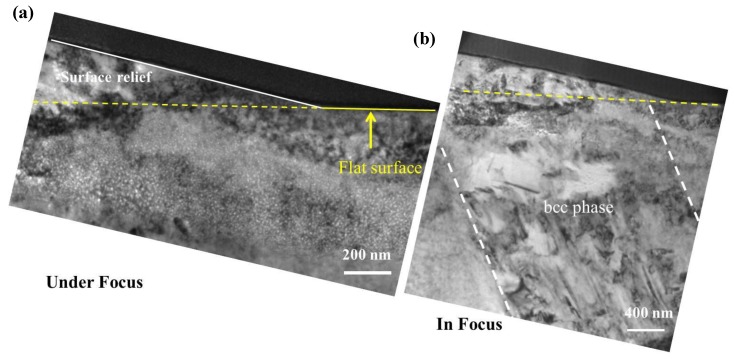
TEM images of the microstructure of the Fe-15Cr-15Ni alloy cryogenically treated at 77 K and heated to 773 K for a 2 × 10^17^ ions cm^−2^ He^+^ irradiation—showing the α′-martensite phase (transformation) that is related to the surface relief at (**a**) high magnification and (**b**) low magnification.

**Figure 10:**
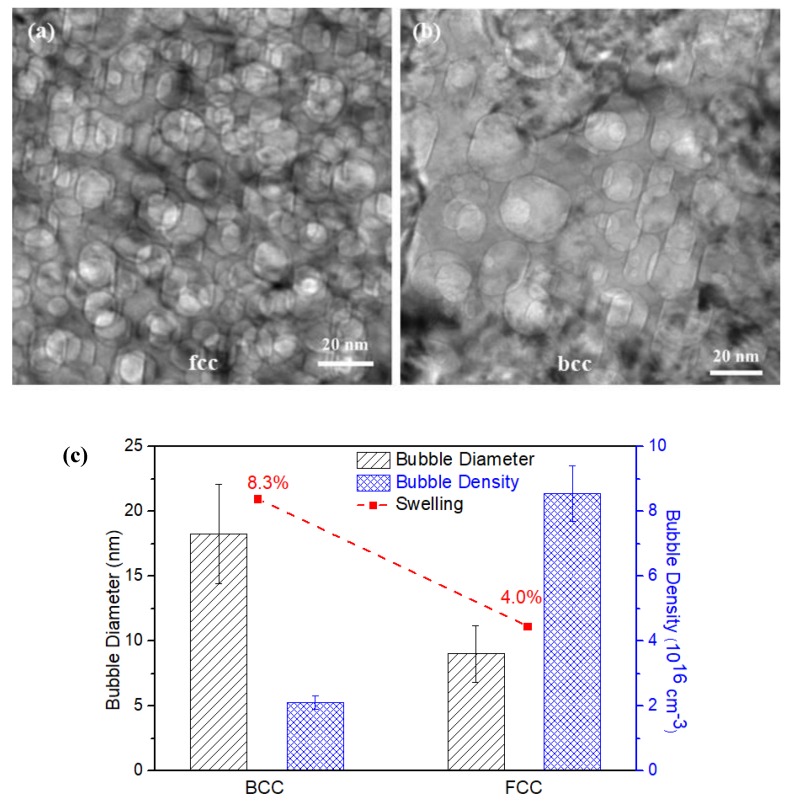
Surface dilation and helium bubbles corresponding to the fcc and bcc phases, (**a**,**b**), respectively. (**c**) Statistical data for the helium bubbles formed in the bcc and fcc phases—including the bubble size, bubble density, and bubble swelling.

Cross-section samples were prepared by the FIB lift-out technique from the surface relief areas. Figure 9 shows the cross-section TEM sample from a surface relief area where a second phase is observed that is consistent with the surface “upheaval” or dilation feature. At higher magnification, Figure 10a,b show helium bubbles at the helium peak concentration region in the fcc and bcc phases. The helium bubbles are much larger but less dense in the bcc phase than in the fcc phase. In order to determine the qualitative effect of the two different phases, the number of the helium bubbles in the peak area was characterized. In the peak helium concentration region, the helium bubbles exhibit the greatest variation in bubble size in the different phases. The plotted results are shown in Figure 10c. The average sizes of helium bubbles in the bcc phase and fcc phases are about ~18 and ~9 nm in diameter, respectively. The density of helium bubbles in the bcc phase is two times lower than that in the fcc phase. The bubble swelling is ~8% in the bcc phase and ~4% in the fcc phase. Because these two areas are adjacent with the same implanted helium concentration, the difference in helium bubble formation can be directly observed and compared. According to the difference in the bubble swelling between the fcc and bcc phases, the surface dilation would only be several nanometers in height. The lattice parameter ratio, $a_{\gamma }/a_{\alpha^{\prime }}$, is 1.244, as calculated from the diffraction pattern in Figure 8b. The “swelling” induced by the bcc phase is about 3.7%, according to the natural lattice differentials. This result cannot yield values of the surface relief that span a range of tens to hundreds of micrometers for our bulk samples. To evaluate the phase transformation contribution to the surface relief, the TEM sample containing both surface “upheaval” and depression was characterized. From the TEM results (Figure 9), it is directly observed that the α′-martensite phase is strongly related to surface relief. 

#### 3.3.3. Helium Bubbles along Phase Boundaries

**Figure 11:**
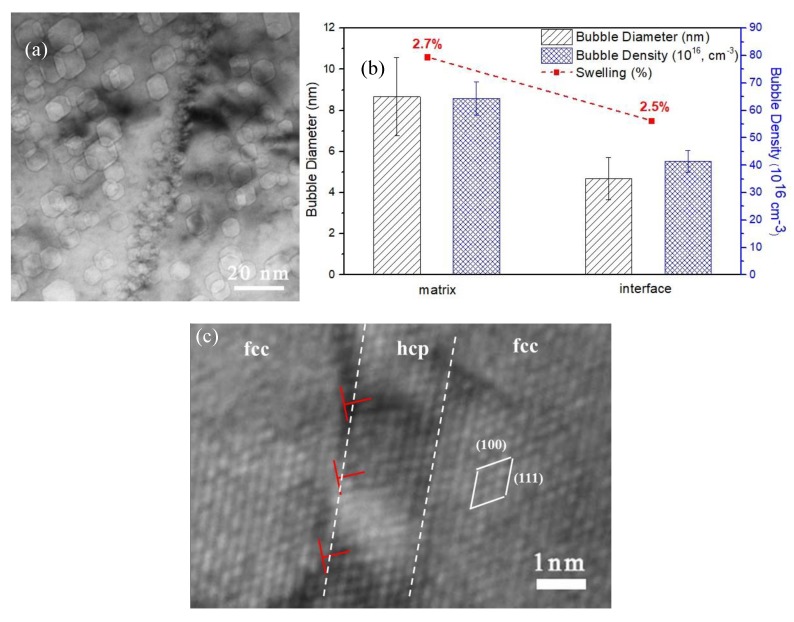
TEM micrograph (**a**) and statistical data (**b**) for the helium bubbles along the fcc–lamellae hcp boundary, including the helium bubble size, helium bubble density, and bubble swelling. (**c**) High-resolution TEM confirms the presence of coherent phase boundaries between the hcp and fcc phases.

As illustrated in Figure 11, a TEM micrograph shows a helium bubble distribution along a coherent twin boundary that has a {111} boundary plane. Figure 11a shows that a large number of helium bubbles with a small size were formed in the vicinity of the coherent fcc–hcp phase boundary after irradiation at 773 K. The distribution of these helium bubbles is roughly uniform, and there are no obvious bubble-denuded zones along the coherent twin boundary. This observation is consistent with previous investigations on irradiated Cu, i.e., the defect agglomerates formed around coherent twin boundaries are similar as those in the interior of the grains. Statistical results are plotted in Figure 11b and the corresponding microstructure is shown in Figure 11a. Compared with the matrix, helium bubbles at the coherent twin boundary are distributed with average diameters that are smaller and the bubble density is higher. 

## 4. Discussion

### 4.1. Phase Transformation in the Fe-15Cr-15Ni Alloy

#### 4.1.1. Reversion of bcc Martensite in Fe-15Cr-15Ni Alloy

As shown in Figure 1, the surface relief spans a range of 100 μm after He^+^ irradiation at 773 K. The same phenomenon was observed by Breedis and Roberson [23]. They reported that the surface relief was produced after reheating the cryo-quenched Fe-16Cr-12Ni sample. Subsequent transformation produces surface upheavals (dilation) or depressions at the identical sites of the previous transformation. West [25] indicated that the martensite reverse transformation that occurs between A_s_ (austenite initiation temperature) and A_f_ (austenite formation final temperature) of Fe-Cr-Ni austenitic alloys is very different depending on the alloy composition. The A_s_ and A_f_ temperatures for the α′ to γ transformation is 813 and 923 K for the Fe-18Cr-8Ni, and 743 K and 883 K for the Fe-18Cr-12Ni composition. The Fe-18Cr-12Ni alloy has the higher Ni content that lowers the M_s_ as well as the A_s_ temperatures [24]. From the previous results, one may speculate that the A_s_ temperature of Fe-15Cr-15Ni should be around 773 K, i.e., the temperature of the He implantation. This means the α′ to γ transformation potentially occurred during the He^+^ irradiation. 

#### 4.1.2. Phases in Cryo-Quenched-Reheated Single Crystal Fe-15Cr-15Ni Alloy

We observed bcc, hcp, and fcc phases in our Fe-15Cr-15Ni alloy. Extensive research has been carried out on the formation of hcp and bcc martensites in austenitic stainless steels, based on the model Fe-Cr-Ni system. In the work of Coleman, West, and Breedis, all three phases were found in a cryo-quenched-reheated FeCrNi alloy [28]. Observations have been reported of reversed austenite nucleating either within the bcc phase or being formed athermally from whole bcc laths. They indicated that two alternative explanations for the appearance of an hcp structure in the Fe-Cr-Ni alloy. One explanation is that the increase in volume due to the formation of the bcc structure produces extensive faulting of the austenite phase. The second possible explanation is attributed to the formation of the hcp structure as an intermediate structure in the transformation sequence.

### 4.2. Helium Bubbles Evolution in fcc, bcc, and hcp Phases

#### 4.2.1. Faceted Bubble Formation

Based on the present observations, a polyhedron composed of {111} planes is expected to surround the bubbles. As noted above, geometric construction of a polyhedron consisting of {111} planes yields an octahedral shape. Nelson determined that the surface energy sequence for fcc iron is *E*(111) < *E*(100) < *E*(110) [29]. In our case, the {100} planes occupy the corners of the octahedron composed of {111} planes, yielding a {111} octahedron with a six {100}-plane truncation. The total surface area of a faceted bubble with the {111} surface decreases when the bubble is truncated by the {100} planes so the truncated octahedron or rounded corners are therefore more energetically favorable.

The bubble formation characteristics in a bcc structure have received relatively less attention. In Figure 8, the faceted helium bubbles are composed of {100}_bcc_ and {110}_bcc_ planes. It is interesting to note that helium bubbles in the lower helium concentration range are elongated along the {100}_bcc_ surface, while they become more “square” in shape in the He peak range. The distinct {100}_bcc_ facets are consistent with the results in the literature [30,31,32,33]. In order to minimize the overall free energy, the facets with the lowest surface energy will occupy most of the bubble surface, and in the bcc structure, the low surface energy is {100}, {110}, and {111}.

Helium bubbles in the hcp phase have a polyhedral geometry bounded by {0001}, {0111}, and {0110} facets, as determined by viewing the voids along the <2110> zone axes. Given the fact that the surface energies of the {0001} and {0111} surfaces are similar, and slightly lower than that of {0110} [34], the equilibrium bubble shape should be approximately equiaxed [35].

#### 4.2.2. Helium Bubble Size in the bcc and fcc Phases

In the present study, the helium concentration and thermal treatment were the same in the bcc and fcc phases. The most important factor that affects the helium bubble nucleation and growth should be the helium atom diffusion process. Because helium has an extremely low solubility in metals, its diffusion is a basic requirement for bubble nucleation and growth [36]. It is the result of random jumps of helium atoms from one stable lattice site to another or diffusion of helium as interstitials. The dominant positions for helium atoms in a lattice are interstitial and substitutional sites, and the preferential position and dominant migration mode depend on the temperature as well as on the irradiation conditions. For helium diffusion under ion irradiation, atomic displacements and resulting vacancies, self-interstitial atoms (SIAs), and clusters of these defects play an important role. Singh also indicated that the dominant mechanism of helium atom diffusion is the “replacement mechanism” between 0.2 and 0.5 T_m_ [3]. Bubble growth by the dissolution and re-trapping mechanism requires the transport of helium atoms and vacancies. Variation of the size, $\bar{r}$, and density, ρ, of bubbles as a function of the annealing time is described by the relations [37]:

(1) $$\bar{r}\sim {\left(D_{He}\right)}^{1/n},$$

(2) $$\rho \sim 1/D_{He},$$

where $D_{He}$ is the diffusion mobility of the helium in the matrix, and the exponent, n, depends on the bubble-growth mechanism (n = 2~6 [38]). In the present study, the temperature of helium implantation was at 773 K (0.2 T_m_ < 773 K < 0.5 T_m_), which corresponds to the application range of this mechanism (i.e., dissolution and re-trapping). 

An analysis of Equations (1) and (2) above shows that as bubbles grow by the dissolution and re-trapping method, the smaller the value of $D_{He}$, the smaller the bubbles and the higher their density should be. Other researchers have indicated that the diffusion coefficient of helium atoms in bcc steel is higher than that in fcc steel by several orders of magnitude when it is in the intermediate temperature range (600 K < T < 1200 K) [39]. This is comparable to the results in our study where the helium bubbles in the fcc phase are larger and their density is smaller than in the bcc phase. Bubble nucleation, under identical irradiation and annealing conditions, occurs in fcc metals much earlier than in bcc metals [38,40], and during He^+^ implantation, a helium atom injected into an Fe lattice would be expected to be located at an interstitial (i.e., tetrahedral) site. 

#### 4.2.3. Phase Transformation Comparison with Helium Bubble Swelling

Reversion of the austenite phase in a Fe-15Cr-15Ni alloy, as pointed out above, induces surface relief features up to several micrometers in size. According to the measurement and calculation of helium bubble swelling effects, e.g., ~8% in the bcc phase and ~4% in the fcc phase in Figure 9c, the bubble swelling-induced changes on the surface relief height would only be about 16 and 8 nm in the bcc phase and the fcc phases, respectively, due to the limited depth range affected by the helium implantation (e.g., up to ~800 nm as shown in Figure 4). Thus, bubble swelling in both bcc and fcc phases is not the primary reason responsible for the measured surface relief in the sample studied here. However, “swelling” induced by the bcc phase transformation is only about 3.7%, based on the lattice differentials. Therefore, the bubble-produced swelling would be much more significant than the surface relief caused by the phase transformation if the helium concentration were constant throughout the sample thickness.

### 4.3. Helium Bubbles Evolution along the Phase Boundary with Misfit Dislocations

Grain/phase boundaries are considered as effective sites for trapping helium. Generally, possible effects of helium bubble formation on mechanical properties are embrittlement where intergranular fracture is induced by helium bubbles’ growth and coalescence. In the past decades, people have made the effort to find interfaces that are excellent in trapping helium and susceptible to helium embrittlement simultaneously.

In this work, as well as some previously reported investigations [41,42], it is clear that the misfit dislocation structure plays a crucial role in helium nucleation. Helium bubbles on the phase boundary with misfit dislocations, described above, are smaller and denser. These observations indicate that boundaries with misfit dislocations are the preferred trapping sites for helium and will also induce helium dispersion. In the investigation of helium at grain boundaries in austenitic steels, Lane and Goodhew [41] also found that helium bubble densities were greatest at grain boundaries containing misfit dislocations and that these dislocations were, in fact, preferred bubble nucleation sites.

Based on the previous investigations [43] and this study, we may begin to envision new ways for how interfaces may be engineered to control the properties of helium implanted into structural materials. For example, by controlling the distribution of phase boundaries with misfit dislocation, helium bubbles or bubble nuclei may be templated with desired nucleation sites, bubble density, and size.

Helium is highly mobile in most metals [44,45], and is an important factor on controlling the voids’ nucleation and growth. When atomic displacement occurs, both helium atoms and the radiation-induced defects are essential for the formation and growth of helium cavities [46]. Wang reported the influence of the helium concentration on the void formation of ferritic martensitic steels under very high radiation damage (up to 500 dpa) and their work also indicated that at certain helium concentrations, helium can suppress void swelling [47]. This is caused by trapped helium or the helium–vacancy complex serves as the nucleation site for voids. Wei reported that in a fcc/bcc multilayer system, the change in hardness is negligible when the layer thickness is less than 10 nm following helium implantation [9]. If the helium or helium–vacancy cluster can be dispersed by tailoring material with misfit phase/grain boundaries, it may effectively suppress the void growth. This may be a new method to design nuclear materials with high helium tolerance and less helium embrittlement bias. 

## 5. Conclusions

(1) Single crystals of a 70Fe-15Cr-15Ni alloy transform from a fcc- to a bcc-structured martensitic phase on cooling to the liquid nitrogen temperature and a reverse transformation when the temperature was returned to room temperature and continued to evolve during He^+^ ion irradiation at 773 K along with an accompanying intermedium phase of the hcp structure. The reverse transformation also induced significant surface relief.

(2) The size of the ion implantation-induced helium bubbles and the corresponding swelling of the bcc phase were significantly larger than that in the fcc phase at the peak helium concentration depth. The helium diffusion coefficient in the bcc phase was higher than that in the fcc phase by several orders of magnitude at the irradiation temperature (773 K). This lead to faster bubble growth in the bcc phase.

(3) Compared to the volume change induced during the phase transformation (fcc to bcc), the bubble-induced swelling was actually much more significant even though the surface relief caused by the phase transformation in the samples investigated in this study was dominant because of the limited depth of the helium implantation.

(4) The faceted bubbles preferred to grow in a shape consisting of the most closely packed planes in both the fcc and bcc phases where the surface energy is a minimum, consistent with previous experimental observations.

(5) Helium bubbles formed along the “plate-like” intermediate hcp phase and its boundary with the fcc matrix were smaller and had a higher density compared to that in the matrix. The misfit phase boundaries can be an effective site for the dispersion of helium atoms, and this property may help to increase the swelling resistance of the material.
